# Complete coding sequence of an Aedes flavivirus strain isolated from *Aedes albopictus* collected in Northern Italy

**DOI:** 10.1128/mra.00146-24

**Published:** 2024-06-25

**Authors:** Essi M. Korhonen, Phuoc T. Truong Nguyen, Giulia Faolotto, Maija T. Suvanto, Anna Maria Nicosia, Maria Grazia Crobu, Ivan Grasso, Olli Vapalahti, Teemu Smura, Paolo Ravanini, Eili Huhtamo

**Affiliations:** 1 Department of Virology, Medicum, University of Helsinki, Helsinki, Finland; 2 Laboratory of Microbiology and Virology, University Hospital Maggiore Della Carità Di Novara, Italy, Piemonte, Novara; 3 Department of Veterinary Biosciences, University of Helsinki, Helsinki, Finland; 4 Department of Virology and Immunology, Helsinki University Hospital Diagnostic Center, Helsinki, Finland; Katholieke Universiteit Leuven, Leuven, Belgium

**Keywords:** *Orthoflavivirus*, Aedes flavivirus, virus-host interactions, Italy, *Aedes albopictus*, RNA virus

## Abstract

Complete genome data for the globally distributed Aedes flavivirus (AEFV) is scarce. We analyzed a new Italian AEFV strain isolated from *Aedes albopictus*. The results demonstrated genetic diversity among Italian AEFVs. The high similarity between AEFV genomes across geographically distant regions suggests long distance spreading via invasive host mosquito species.

## ANNOUNCEMENT

Aedes flavivirus (AEFV) belongs to the classical insect orthoflavivirus clade (*Flaviviridae*) that does not infect vertebrate or mammalian cells (1, 2). It was first isolated from *Aedes albopictus* and *Aedes luteocephalus* mosquitoes in Japan in 2003 (1), after which it has been detected from various geographical locations and mosquito species (3–7). As of 18 November 2023, only six coding-complete sequences are available in the NCBI GenBank database despite being repeatedly detected in pan-flavivirus screenings of Italian mosquitoes (5, 8–10).

Following a flavivirus screening study, we attempted to isolate AEFV from an *Ae. albopictus* homogenate sample that was AEFV-positive using pan-flavi Reverse-transcription PCR (RT-PCR) and Sanger sequencing (11). The specimen was collected near Sasso Marconi, Bologna, Italy (44°23’42.6”N 11°15’15.8”E) on 9 July 2011. A mild cytopathic effect was seen on C6/36 cells after 5 days subsequent to adding the mosquito homogenate to the cells. The harvested supernatant sample was used for RNA extraction using TRIZOL reagent and Next-generation sequencing (NGS) library preparation using NEBNext Ultra II RNA library prep kit as previously described (12).

The library was sequenced using Illumina NovaSeq 6000 system and SP Reagent Kit v.1.5 (500 cycles). The reads were quality filtered, *de novo* assembled, and annotated using fastp (13), MEGAHIT (14), and SANS-parallel algorithms (15), respectively, implemented in Lazypipe (16, 17), followed by re-mapping the sequence reads using a *de novo* assembled AEFV contig as a reference with BWA-MEM algorithm (18) implemented in HAVoC (19). This resulted in 1,294,614 paired-end reads, and 1,239,094 quality-filtered (Q30) reads, of which 728,908 mapped to AEFV. The mean read coverage was 8,767.

**Fig 1:**
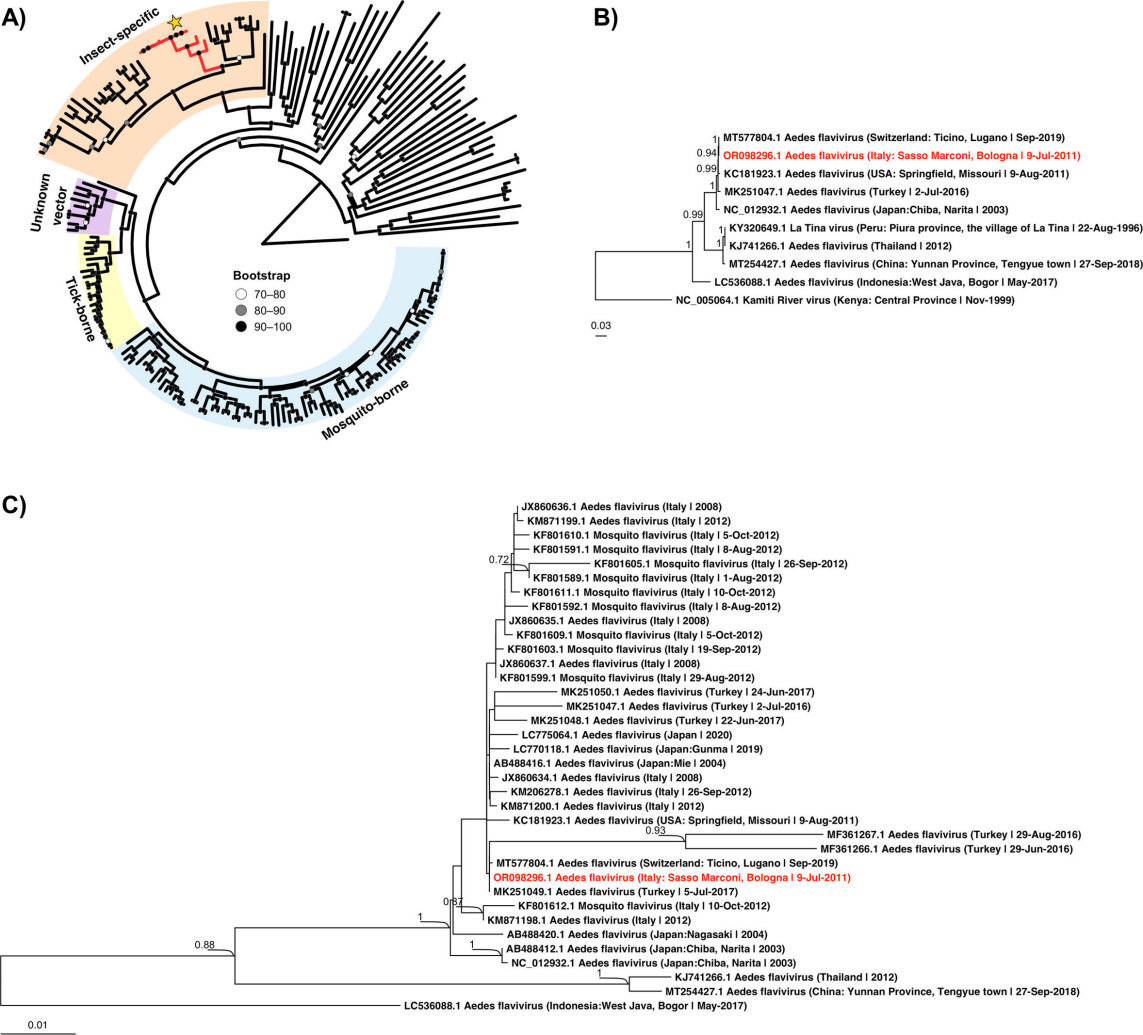
Maximum-likelihood trees depicting the evolutionary relationship of AEFV strain Sasso Marconi 2011. (**A**) Phylogenetic tree of *Flaviviridae* polyproteins (*n* = 193) and rooted with bovine viral diarrhea virus 1 (*Pestivirus*). The yellow star indicates the position of our AEFV strain. (**B**) Tree based on open reading frames using nucleotide sequences of closely related viruses highlighted in red in panel A (*n* = 10) and rooted with the orthoflavivirus, Cell fusing agent virus (CFAV). It was hidden in the final tree for readability. (**C**) Tree of NS5 nucleotide sequences (*n* = 36) that were ≥300 bp long and had an identity of ≥80% to the NS5 sequence of our AEFV strain according to BLASTn. The tree was also rooted with CFAV and hidden for readability. The sequences in all trees were obtained from NCBI GenBank. These were aligned with MAFFT v.7.520 (23) and the alignments were used to compute the maximum-likelihood trees with 1,000 bootstraps each. The tree in panel A was computed using IQ-TREE2 v.2.1.2 (24) with the best-fit amino acid substitution model LG + F + R9 determined by the integrated ModelFinder (25), and the trees in panels B and C using MEGA11 (26) with the general time reversible (GTR) model, uniform rates, using all sites, extensive subtree-pruning-regrafting (SPR level 5), default setting for making the initial tree, and no branch swap filter. Only bootstrap values of ≥0.7 are shown in the final trees.

The obtained complete coding sequence was 11,065 base pairs long with 49.78% GC content in the coding region of the polyprotein and the strain was designated as Sasso Marconi 2011 (GenBank accession: OR098296). It represents the second coding-complete AEFV genome from Europe, the first being obtained from Switzerland (20). These two sequences were nearly identical sharing 99.93% nucleotide similarity in the open reading frame (ORF; Table 1) according to Clustal Omega v.1.2.3 (21). Based on partial NS5 coding region (362 bases), our isolate was identical to a Turkish strain 20AEFV (GenBank accession: MK251049). Phylogenetic analysis suggested that the strains from different countries were closely related without clear geographical clustering (Fig. 1). Our isolate clustered together with the Swiss strain AEFV/Ticino/2019 (GenBank accession: MT577804) based on the ORF (Fig. 1B). The lack of geographical clustering with AEFV sequences corresponds with the invasive nature and wide dispersion pattern of *Ae. albopictus* (22). Whether the currently observed AEFV diversity in some areas is due to *Ae. albopictus* re-introduction events requires further study.

**TABLE 1:** Nucleotide identity and GC content comparison of AEFV strain Sasso Marconi 2011 to other AEFVs and closely related insect-specific orthoflaviviruses.

Virus	GenBank accession	Capsid	Membrane	Envelope	NS1	NS2A	NS2B	NS3	NS4A	NS4B	NS5	Average identity	ORF	GC% (ORF)
Aedes flavivirus strain Sasso Marconi 2011	OR098296	100.00	100.00	100.00	100.00	100.00	100.00	100.00	100.00	100.00	100.00	100.00	100.00	49.78
Aedes flavivirus strain AEFV/Ticino/2019	MT577804	100.00	100.00	100.00	99.74	100.00	100.00	99.89	99.75	100.00	99.96	99.93	99.93	49.76
Aedes flavivirus strain AEFV-SPFLD-MO-2011-MP6	KC181923	99.70	98.92	99.61	99.23	99.84	99.55	99.26	98.77	99.48	99.66	99.40	99.47	49.91
Aedes flavivirus strain KRD32	MK251047	-^*a*^	99.46	99.69	99.15	99.84	98.21	99.09	99.26	99.61	-^*a*^	99.29	-^*a*^	–^*a*^
Aedes flavivirus strain Narita-21	NC_012932	98.50	98.39	98.30	98.89	99.19	98.66	98.64	98.02	98.05	98.72	98.54	98.57	50.05
Aedes flavivirus strain Bangkok	KJ741266	93.39	93.55	88.66	89.74	96.14	94.18	90.74	88.64	92.48	91.37	91.89	91.14	49.93
La Tina virus isolate 49_LT96	KY320649	93.39	93.55	88.66	89.74	96.14	94.18	90.74	88.64	92.48	91.37	91.89	91.14	49.91
Aedes flavivirus strain TC4A8_18–9L-Y-T-Aea-B-1–1	MT254427	93.09	91.94	88.73	89.74	96.30	93.96	90.97	88.40	92.61	91.33	91.71	91.15	49.83
Aedes flavivirus AEFV/MQ/29/Bogor/2017	LC536088	88.29	87.63	88.50	89.32	95.17	91.05	87.39	91.85	87.42	88.81	89.54	89.03	49.10
Kamiti River virus	NC_005064	47.15	51.61	59.65	60.85	55.07	50.78	61.61	52.59	54.60	66.18	56.01	59.50	49.99
Cell fusing agent virus strain Galveston	NC_001564	24.32	39.88	35.04	54.10	55.23	50.56	60.31	48.40	54.73	65.73	48.83	53.45	50.99

^*a*^ Values that could not be computed due to unavailable or partial sequences.

## Data Availability

The AEFV 2011 Sasso Marconi strain genome has been deposited in NCBI GenBank database under accession no. OR098296. The reads of the virus are available in NCBI Sequence Read Archive (SRA) database with the accession no. PRJNA1050177.
